# Rural population’s preferences matter: a value set for the EQ-5D-3L health states for China’s rural population

**DOI:** 10.1186/s12955-022-01917-x

**Published:** 2022-01-29

**Authors:** Gordon G. Liu, Haijing Guan, Xuejing Jin, Han Zhang, Samantha A. Vortherms, Hongyan Wu

**Affiliations:** 1 National School of Development, Peking University, Beijing, 100871 China; 2 Institute for Global Health and Development, Peking University, Beijing, 100871 China; 3 Department of Pharmacy, Beijing Tiantan Hospital, Capital Medical University, Beijing, 100070 China; 4 Centre for Evidence-Based Chinese Medicine, Beijing University of Chinese Medicine, Beijing, 100029 China; 5 Department of Global Health and Population, Harvard T.H. Chan School of Public Health, Boston, MA 02215 USA; 6 Department of Political Science, University of California, Irvine, CA 92697 USA; 7 School of Medicine and Health Management, Guizhou Medical University, Guiyang, 550025 China; 8 Key Laboratory of Environmental Pollution Monitoring and Disease Control, Ministry of Education, Guizhou Medical University, Guiyang, 550025 China

**Keywords:** EQ-5D, China, Rural resident, Time trade-off, Quality of life, Value set

## Abstract

**Purpose:**

To develop an EQ-5D-3L social value set based on Chinese rural population’s preferences using the time trade-off (TTO) method, and to compare the differences in preferences on health states between China urban and rural population.

**Methods:**

Between Sep 2013 and Nov 2013, a total of 1201 participants were recruited from rural areas of five Chinese cities (Beijing, Chengdu, Guiyang, Nanjing, and Shenyang) using a quota sampling method. Each respondent valued 13 health states using the TTO, and a total of 97 EQ-5D-3L health states were directly valued for estimating the value set. Various models with different specifications were explored at both aggregate and individual levels. The final model was determined by a set of predefined selection criteria.

**Findings:**

An ordinary least square model at the aggregate level included 10 dummy variables for specifying the level 2 and 3 for each dimension and an N3 term presenting any dimension on level 3 was selected as the final model. The final model provides a value set ranges from − 0.218 to 0.859. The predicted utility values were highly correlated with but consistently lower than that of the published Chinese EQ-5D-3L value set (for urban population).

**Conclusion:**

The availability of the China rural value set provides a set of social preferences weights for researchers and policy decision-makers for use in China rural area.

**Supplementary Information:**

The online version contains supplementary material available at 10.1186/s12955-022-01917-x.

## Key points for decision makers

The socioeconomic status and demographic characteristics vary between urban and rural population in China. This study is the first attempt to provide a value set of weights for health states based on the preferences of Chinese rural registrants, and offers an evidence-based approach to health utility measurement in policy decision-making for urban–rural health care system integration and health equity promotion as an important supplement to the Chinese urban populations.

## Background

Health utility (people’s preference towards a particular health outcome) is used to calculate quality-adjusted life years (QALY) in cost-utility analysis (CUA) which allows comparison across different health programs and can provide compelling evidence for medical decision-making [1]. However, preferences on health states are not universal across jurisdictions [2], even vary among subpopulations in a jurisdiction [3, 4], many jurisdictions recommend the preference from a representative sample be used to develop the value set of utility-based instruments such as the EuroQol Group’s EQ-5D which is one of the most widely used generic measures. Thus, health utilities derived from value sets contextualizing the indirect preference-based instruments should be based on locally defined weights to ensure valid inference [4–6]. Using a value set derived from an unrepresentative sample in medical decision-making may lead to inequality and unethical consequences.

In China, residents in rural areas accounted for more than 50% of the whole Chinese population before 2011 [7]; since then, with the continuous advancement of China’s urbanization process, the number of rural residents has declined, but still accounts for a considerable proportion. In 2020, the proportion of residents in China’s rural areas was around 40% of the whole population [8].

The socioeconomic status, demographic characteristics, lifestyles and health conditions vary between urban and rural population in China. More specifically, from the perspective of income, between 2013 and 2020, the annual per capita disposable income of the urban population (43,834 RMB ≈ 6838 USD in 2020) in China was approximately three times that of the rural population (17,131 RMB ≈ 2673 USD in 2020) [7]; in rural areas, the number of people receiving minimum living security benefits is 4.5 times that of urban people [8]. For the education level, the proportion of the Chinese rural population with a high school or higher education is about 10%, and this proportion exceeds 38% in urban areas [9, 10]. For family structure, on average, each household in the rural areas has one more person than each household in urban areas [9]. In addition, the proportion of people over 60 years old in rural areas is 15.0%, while in urban areas is 11.7%, and the total dependency ratio is 51.8% in rural areas and 34.7% in urban areas [9]. For the population-level health status, the highest mortality disease among rural residents is heart disease (164.66/100,000), while the highest mortality disease among urban residents is cancer (161.56/100,000) [11]. The proportion of people over 60 who self-reported unhealthy or unable to take care of themselves in rural and urban areas were 20.3% and 12.3%, respectively [9].

EQ-5D is the most commonly used generic preference-based instrument in economic evaluations around the world. It has five dimensions including mobility, self-care, usual activities, pain/discomfort, and anxiety/depression. For the 3L version (EQ-5D-3L), each dimension contains three levels of severity i.e., no problems, some/moderate problems, and extreme problems, which can generate a total of 243 (= 3^5^) unique health states ranging from 11111 (full health) to 33333 (the worst health state). For the 5L version, each dimension has five response levels.

*China Guidelines for Pharmacoeconomic Evaluations (2020)* recommends that in CUA, researchers should use generic preference-based instruments which have a value set based on the Chinese population’s preferences [12]. The instruments that meet the above requirements in China are EQ-5D-3L, EQ-5D-5L and SF-6D V2 [12]. The EQ-5D-3L (Liu et al. 2014) and EQ-5D-5L (Luo et al. 2017) Chinese value sets that endorsed by the EuroQol group were only based on the urban population’s preferences [13, 14]. Zhou and colleagues published another Chinese EQ-5D-3L value set based on an urban and rural mixed sample; however, non-standard time trade-off (TTO) method was employed in this study, that is, an open-ended TTO question rather than an iteration-based procedure was used, in addition, both the dead and the concept of worse than dead health states were not used in the elicitation procedures [15]. Non-standard TTO measurement methods may cause deviations from respondents’ true preference for health states that be valued. Value sets estimated based on such methods may also be biased, which would lead to bias in CUA.

Previous studies have shown that respondents with different characteristics have different preferences for the same health states. These characteristics include age, gender, educational level, marital status, economic status, health conditions, belief in life after death, and attitudes towards whether bad living is better than good death [2, 15–19]. In addition, the area where the respondent lives also affects their preference for health states. Zhou et al. found that there is a difference in preference for the same health states between rural and urban populations in China, and this difference still exists even after controlling for age, gender and economic status [15]. In addition, the distribution of economic resources, medical materials, and educational resources in rural and urban areas in China is uneven [7, 9–11], and some health assistance policies are also specifically targeted at rural area. Therefore, using existing value sets to support health care resources allocation in the rural areas may cause inequity or improper decisions. Therefore, a value set of EQ-5D based on the Chinese rural population’s preferences is warranted. Such a value set can help researchers better explore and understand the differences in preferences on health outcomes between China rural and urban populations, and can also provide some support for decision-makers in avoiding inequity or improper decisions.

It is admitted that the EQ-5D-5L has demonstrated better measurement properties than the EQ-5D-3L in many populations [20, 21], considering the relatively lower education level and larger proportion of older people of the rural population, less response levels may help people to better understand the TTO tasks and the instrument per se in valuation studies and health surveys in rural areas [22, 23]. The present study primarily aimed to establish an EQ-5D-3L value set based on a sample which can represent the China's rural population. The secondary objective was to compare the differences in preferences on health states between China urban and rural population.

## Methods

### Study overview

The China Rural population EQ-5D-3L valuation study was conducted between September 2013 and November 2013, and was carried out in rural areas of five Chinese cities including Beijing, Chengdu, Guiyang, Nanjing, and Shenyang. Data were collected through face-to-face interviews. Paper based traditional TTO method was used as the main preference elicitation technique.

### Sampling

From the highest to lowest, there are three hierarchical levels in Chinese rural area, i.e., county, township, and village. Participants were recruited from the five cities’ rural areas using quota sampling in terms of age and sex according to the Sixth National Population Census. Within each city’s rural area, 3 geographically dispersed counties were selected. Within each county, respondents were recruited from at least two townships’ village area. In order to ensure geographic distribution of the sample, no more than 40 respondents were recruited from the same township. In addition, in order to avoid mutual influence of preferences on health states among household members, no more than one participant per household was selected. The target sample size of each city’s rural area was 240 respondents.

Participant inclusion criteria were as follows: (1) were rural Hukou holders; (2) had inhabited the selected village for at least six months; (3) aged 16 years or older; (4) were able to read in mandarin; (5) did not have any cognitive impairment and mental disease; and (6) did not have any serious vision, hearing and communication problems. During the interview, if an interviewee could not understand the survey in any aspect, the interviewer terminated the interview.

### Health state selection

We followed the Paris protocol and proposed a direct valuation of a total of 97 impaired EQ-5D-3L health states (included “33333”), to estimate the value sets. Except for “33333”, the 96 selected health states were categorized into three levels: mild, moderate, and severe. Mild states were those health states have no level 3 on any dimension and only have level 2 on up to three dimensions; severe states had no level 1 on any dimension; all other health states were considered moderate (more detailed information on selecting health states was described in the revised MVH protocol [24]). A total of 24 mild, 48 moderate and 24 severe states were randomly assigned into eight fixed blocks. Each block included 12 health states with three mild, six moderate, and three severe states. In addition, states 11111, 33333, and death were assigned to each block, making a total of 15 health states in each (Table 1). Health state blocks were randomly assigned to each participant during the interview.

**Table 1:** EQ-5D-3L health states distributions

Severity	Group							
	A	B	C	D	E	F	G	H
Mild	11112	11221	11212	22211	21111	12221	22111	11121
	12122	11222	12211	21211	22121	21121	22112	21122
	12112	11122	12121	11211	21112	21221	12212	12111
Moderate	31213	11313	12123	21332	21331	11332	12312	23321
	31311	32123	21313	21133	11123	13222	13211	21231
	23132	21311	12313	33122	22232	12331	23311	11232
	21123	11323	23313	22221	11312	33221	32111	21312
	23231	33121	33313	13123	13232	31222	22313	31131
	22113	33211	33231	11223	23222	33312	23131	31313
Severe	22233	23233	33332	33233	33223	32332	33232	22333
	22332	23322	32322	23323	33323	23223	23332	22323
	23333	33222	32223	33322	32233	32333	32323	32232
Others	11111	11111	11111	11111	11111	11111	11111	11111
	33333	33333	33333	33333	33333	33333	33333	33333
	Death	Death	Death	Death	Death	Death	Death	Death

### Data collection

The interview process included the following tasks: (1) assessing respondent self-reported health state using the Chinese version of EQ-5D-3L instrument; (2) ranking the 15 health states in terms of their severity; (3) rating the 15 health states using a vertical hash-marked visual analogue scale (VAS); (4) evaluating the same set of health states using the TTO except for 11111 and death; (5) collecting participant’s socio-demographic information.

Each health state was described on a separate card, and the level of severity was marked by different colors: green, yellow, and red indicate no problem, moderate, and severe problem, respectively. In step 2 and 3, each health state had a 10-year duration followed by death. A double-side time board was used in step 4, the TTO exercise, to illustrate the different lengths of hypothetical lives, with one side representing states better than death and the other side representing states worse than death [25]. In order to minimize the memory effect, the cards were reshuffled at the beginning of each step.

In step 4, each TTO task started with asking participant whether health state be valued was better or worse than or equals to death. If the state was considered better than death, then the iteration process was trading off *t* years in state 11111 (Life A) against the 10 years in the being valued health state (Life B). If the state was considered to be worse than death, the participant was asked to compare living in the target state for (10 − *t*) years followed by *t* years in the state 11111 (Life A) and immediately death (Life B). The minimal changeable unit in the iteration process was 6-month. If the state was considered as equivalent to death, the valuation of this state is completed and the interviewer would move on to the next one. The TTO utility score (U) was calculated as *t*/*10*, − *t/*(*10* − *t*) and 0 for better than death states, worse than death states and equivalent to death states, respectively, where the *t* is life years in the state 11111 when Life A and Life B were considered about the same.

A total of 61 graduate students and faculties were recruited as interviewers from one university in each one of the five cities and trained by Peking University’s China Center for Health Economic Research (CCHER). All the interviewers participated in a three-day standardization training session, where they were trained to grasp the skills and procedures for interviewing through mock exercises and were required to accomplish at least one pilot interview in the selected city.

### Data analysis

#### Data logic and transformation

In line with the previous studies [14, 26], data exclusion criteria were: (1) data missed for all; (2) only 1 or 2 states were valued; (3) all states were reported with the same value; (4) all states were valued as worse than death; and (5) had logical inconsistencies for 4 or more pairs of states. Logical consistency was defined as: for a given pair of health states, if at least one dimension of state A (such as 11121) is better than the corresponding dimension in state B (such as 11123) and other dimensions are not worse than their counterparts in state B, then the valuation for the state A should be at least as good as the valuation of the state B.

#### Modeling of TTO values

By design, the TTO utility values originally ranged from − 19 to 1 [27]. In order to eliminate the outlier effects of extremely low values in subsequent analyses, a linear transformation formula applied in a previous Chinese study [14], was used to rescale the negative values to range from − 1 to 0 (*U*^∧^ = *U*/*19*). State 11111 and death were anchored as 1 and 0, respectively.

The dependent variable of all models was disutility, which was defined as 1 minus the TTO value. The main effects model included only 10 dummy variables present level 2 and level 3 problems for each one of the five dimensions. The following interaction terms were considered in our study: (1) N3 term, which equalled to 1 if the health state being valued included at least 1 dimension at level 3, otherwise equalled to 0; (2) D1 term, which was the number of dimensions with problems beyond the first one; (3) I2 and I3 terms, which were the number of dimensions at level 2 and level 3 beyond the first one, respectively; and (4) I2sq and I3sq terms, which were the square I2 and I3, respectively. The N3 model contains one interaction variable of N3 term [27]; while the D1 model contains five interaction variables of D1, I2, I3, I2sq and I3sq [28].

To estimate the TTO values on all health states, models at both aggregate and individual levels were constructed. At the aggregate level of analysis, the mean of the TTO values was used to summarize the value of each health state. Both ordinary least square (OLS) and weighted least square (WLS) regression were employed, with the number of respondents who rated a particular health state as the weight. At the individual level of analysis, Pooled OLS, fixed effect/random effect estimation model were taken into account.

The Breush–Pagan test was performed to test for heteroscedasticity and the Jarque–Bera test evaluated whether the residual term in the regression models had skewness and kurtosis consistent with abnormal distributions. A Hausman’s test was used to decide between random effects and fixed effects models. The Ramsey Regression Equation Specification Error Test (RESET) for model misspecification was also examined.

#### Model selection

Four criteria were used to select the final model for the value set: (1) logical consistency; (2) sign and significance of regression coefficients: the coefficients of main effects should be statistically significant and positive, and the coefficient of level 3 was expected to be higher than that of level 2 for each dimension; (3) goodness of fit, the mean absolute error (MAE) and the root mean squared error (RMSE) were calculated, and the number of health states that had an absolute error greater than 0.025, 0.05 and 0.10 was estimated; and (4) parsimony, if several models performed similarly to the criteria specified above, the most parsimonious model would be preferred.

The robustness of the final model was assessed by using a split-half strategy: a subset of two-thirds of the observations was randomly selected and used to re-estimate the model [27]. The estimated coefficients were then used to generate predicted values, which were then compared with the observed values of the remaining one-third of observations. The Pearson’s product-moment correlation coefficient between the observed and the predicted values of health states is presented. In addition, a leave-a-state-out cross validation approach was used by excluding each health state in turn from estimating the value set and then calculating the MAE in predicting that omitted health state [29].

#### Comparison with the urban value set

The preference of five dimensions in the allocation of disutility was compared among rural and urban registrants in order to analyze which dimension was the leading factor affecting health utility. The Bland–Altman plot was used to assess the difference of predicted utilities estimated from the urban and rural value set. In addition, we also compared the correlation coefficient between the one-third sample’s mean observation values and the rural predicted values and that between the one-third sample’s mean observation values and the urban predicted values.

#### Estimating a value set using combined rural and urban samples

This study adopts the same study design and analysis method as the urban value set developed by Liu et al. [14]. Considering there would be potential end-users interested in urban and rural merged population's preference, we merged Liu's study sample with our sample and estimated a value set using the same data analysis strategy used in the present study.

All statistical analyses were conducted using STATA/SE 15.1.

## Results

### Participants characteristics

A total of 1201 respondents completed the interview. Twenty-eight respondents were excluded due to quality issues (2 respondents gave the same values for all 13 TTO tasks and 1 valued all states worse than death, 25 respondents had four or more logical inconsistencies). As a result, a total of 1173 respondents formed the valuation sample, of which 592 (50.47%) were females. The mean (standard deviation) age was 43.21 (SD = 15.75) years, (more details about the characteristics of participants see Table 2). The study sample was generally representative of the rural Chinese population in terms of gender, age, ethnicity, marital status, with a high over-representation of high educational level population. At the aggregate level, the means of all the 97 health states did not show any logical inconsistency in pairwise comparison.

**Table 2:** Study sample characteristics in comparison with rural Chinese population aged 16 or more

Characteristic	Study sample (N = 1173)	Rural Chinese population aged 16 or more*
*Sex*		
Male	49.53	50.44
Female	50.47	49.56
*Age*		
16–20	8.53	9.34
21–30	16.54	18.13
31–40	19.27	18.79
41–50	21.31	20.31
51–60	17.14	15.98
60 +	17.22	17.45
*Ethnic group*		
Han	84.65	88.65
Minority	14.66	11.27
No answer	0.68	0.07
*Education level*		
Primary and lower	24.21	41.61
High school	68.37	56.01
College and higher	7.42	2.38
*Marital status*		
Unmarried	14.07	15.50
Married	81.42	80.18
Divorced	1.19	1.13
Widowed	3.24	3.19
Other	0.09	
*Having chronic condition*		NA
Yes	25.83	
No	71.70	
Unclear	2.47	
*Working status*		
Formal	13.13	NA
Temporary	10.91	NA
Freelance	17.05	NA
Retired	2.47	1.12
Student	6.31	4.53
Farmer	39.05	NA
Unemployed	10.57	11.43
Other	0.51	NA
*Monthly income*^a^		NA
0–1000	17.14	
1001–5000	53.45	
5001–10,000	16.03	
> 10,000	2.64	
Missing	10.74	
*Self-reported health status*		NA
Very good	25.58	
Good	33.25	
Fair	37.34	
Poor	3.41	
Very poor	0.43	
*EQ-5D-3L any problem*		NA
Mobility	5.29	
Self-care	2.30	
Usual activities	4.43	
Pain/discomfort	20.12	
Anxiety/depression	11.76	

*NA* not available

^a^*RMB* Renminbi, *EQ-5D-3L* three-level EuroQol five-dimensions

*Source: National Bureau of Statistics, the 2010 population census of the People’s Republic of China

### Modeling

At the individual level, the Hausman’s test didn’t reject the null hypothesis of no inconsistencies between coefficients (χ^2^ = 4.39, *P* = 0.9280). Therefore, we only conducted pooled OLS and random effects models rather than fixed effects estimation. However, no model passed the Jarque–Bera test for normality of the residuals (*P* < 0.001). At the aggregate level, the results of OLS and WLS regression are presented. Three types of models passed the all tests, with the exception of the N3 model estimated by OLS regression (Breusch-Pagan test for heteroscedasticity, χ^2^ = 22.91, *P* = 0.0182). It seems that models at the aggregate level performed better.

As shown in Table 3, at the aggregate level (the coefficients estimates and fit statistics results at the individual level of analysis are reported as Additional file 1: Appendix S1), all the estimated coefficients of main effects in each model (10 dummy variables), no matter using OLS or WLS regression, are positive and significant. In N3 model, estimates for the N3 term using OLS regression were significant, reflecting the much greater disutility associated with extreme problems, but this term turned to be insignificant in WLS regression. In contrast, I3, I2 and I2sq terms were insignificant in the D1 model using both of the OLS and WLS regressions (coefficients of the D1 model that only includes significant variables see Additional file 2: Appendix S2).

**Table 3:** Parameter estimates and fit statistics of aggregate level models using OLS and WLS regression

Variable	Main effects				N3				D1			
	OLS		WLS		OLS		WLS		OLS		WLS	
	Coef	SE	Coef	SE	Coef	SE	Coef	SE	Coef	SE	Coef	SE
Constant	0.071	0.007	0.071	0.007	0.067	0.007	0.070	0.007				
MO2	0.102	0.005	0.100	0.005	0.101	0.005	0.099	0.005	0.167	0.007	0.166	0.007
MO3	0.279	0.007	0.280	0.007	0.275	0.007	0.279	0.007	0.365	0.014	0.370	0.014
SC2	0.102	0.005	0.101	0.005	0.103	0.005	0.101	0.005	0.169	0.007	0.169	0.007
SC3	0.242	0.006	0.244	0.006	0.239	0.007	0.243	0.007	0.330	0.015	0.336	0.015
UA2	0.087	0.006	0.085	0.006	0.086	0.006	0.084	0.006	0.151	0.007	0.150	0.007
UA3	0.222	0.006	0.223	0.006	0.217	0.007	0.222	0.007	0.308	0.013	0.313	0.014
PD2	0.110	0.006	0.110	0.006	0.110	0.006	0.109	0.006	0.175	0.007	0.175	0.007
PD3	0.237	0.006	0.240	0.006	0.232	0.007	0.239	0.007	0.323	0.014	0.329	0.014
AD2	0.075	0.005	0.074	0.005	0.074	0.005	0.073	0.005	0.139	0.009	0.138	0.009
AD3	0.177	0.006	0.180	0.006	0.172	0.007	0.178	0.007	0.262	0.014	0.267	0.014
N3					0.016^‡^	0.009	0.005^§^	0.009				
D1									− 0.073	0.013	− 0.077	0.014
I2									0.009^§^	0.017	0.015^§^	0.018
I2sq									− 0.000^§^	0.003	− 0.000^§^	0.003
I3									− 0.022^‡^	0.013	− 0.028^†^	0.013
I3sq									0.001^§^	0.003	0.003^§^	0.002
*Fit statistics*												
Adjusted R^2^	0.993		0.995		0.993		0.995		0.999		0.999	
MAE	0.018		0.017		0.017		0.017		0.017		0.017	
RMSE	0.024		0.024		0.024		0.024		0.024		0.023	
No. (of 97) > 0.025	28		29		27		28		27		28	
No. (of 97) > 0.05	2		3		2		3		2		2	

*P* < 0.01 and Heteroskedasticity-robust standard error for all regression coefficients unless otherwise stated; there are no health states that had an MAE greater than 0.1 for all models; OLS, ordinary least square; WLS, weighted least square; Coef, coefficient; SE, standard error; MAE, mean absolute error; RMSE, root mean squared error; ^†^0.01 ≤ *P* ≤ 0.05; ^‡^0.05 < *P* ≤ 0.1; ^§^*P* > 0.1

**Fig. 1:**
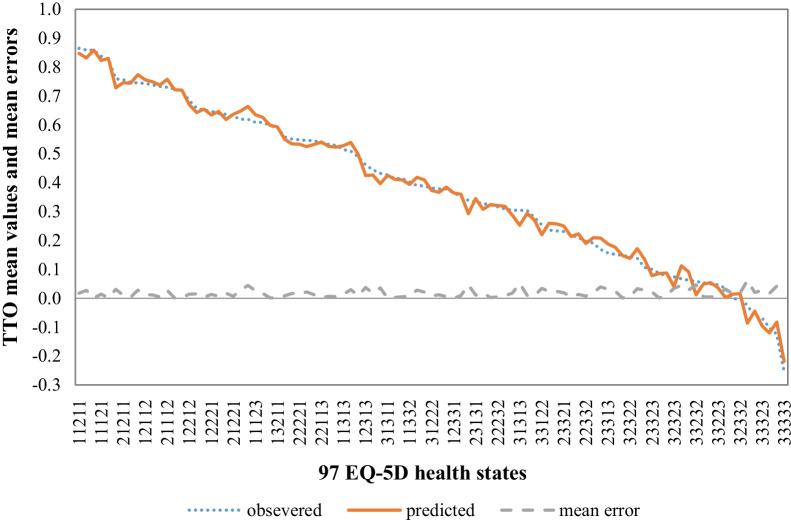
Observed values, predicted values, and mean errors of 97 EQ-5D health states

The results of the main effects, N3, and D1 models from modeling of aggregate level data using WLS regression were generally worse than those based on OLS regression. Specifically, the number of states with an absolute error greater than 0.025 in each model using WLS regression was one more than the number using OLS regression, which potentially resulted from the outweighed number of values for state “33333” (*n* = 1173, SD = 0.41). Finally, the N3 model based on OLS regression with robust standard error to correct for heteroscedasticity without specifying any form for the variance at the aggregate level was selected as the best performing model. The selected model passed the Jarque–Bera test for normality of the residuals (χ^2^ = 5.99, *P* = 0.6582). There was no model or functional form misspecification as suggested by the Ramsey RESET test (F = 0.03, *P* = 0.9916). Figure 1 shows the estimated values plotted against the mean observed TTO values for the 97 health states used in this model. For instance, the value of “23221” was 1 − 0.067 − 0.101 − 0.239 − 0.086 − 0.110 − 0 − 0.016 = 0.381 (see Additional file 3: Appendix S3 to find the utility of 243 health states).

### Robustness

**Fig. 2:**
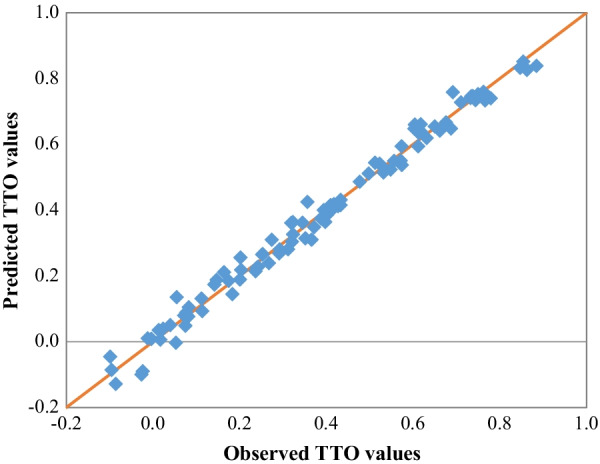
Robustness test: the mean observed and estimated TTO values for the 97 health states

Figure 2 presents each health state’s the mean observed value of the one-third sample and the predicted values for the two-thirds sample in the split-half validation process. The correlation coefficient was 0.9937. In the leave-a-state-out cross-validation for the 97 health states, 33 states (34.0%) had MAE less than 0.01, only 6 states (6.2%) had MAE greater than 0.05, and the largest MAE was 0.071.

### Comparison with urban study

The N3 model based on an OLS regression at the aggregate level turns out to be the best performing model. Of the five EQ-5D dimensions in levels 3, the marginal effect of mobility on health utility is bigger than the other four dimensions in both rural and urban respondents. The dimensions at level 3 exerting the least influence on health utility are different between rural and urban respondents; the former is anxiety/depression, while the latter is usual activities.

**Fig. 3:**
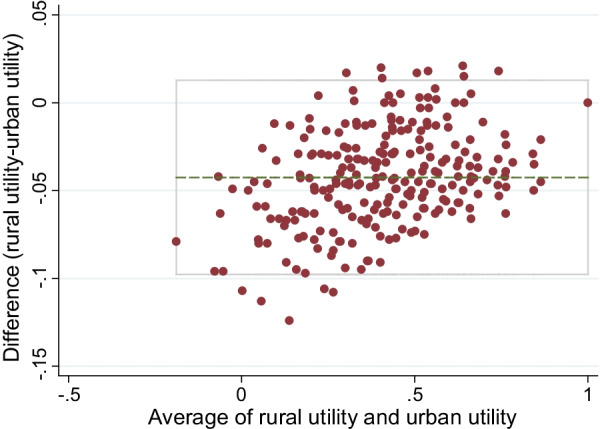
Bland–Altman plots of 243 predicted utilities from rural and urban Chinese population

The Bland–Altman plot indicated that the 95% limits of agreement was − 0.098 to 0.013, and 13 (5.35%) health states utility values exceed the 95% limits of agreement (Fig. 3).

**Fig. 4:**
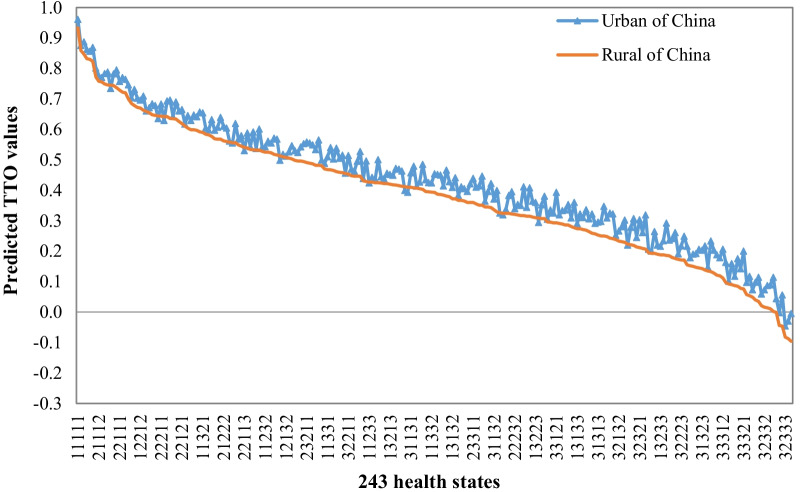
Comparison of utilities based on 243 EQ-5D health states between rural and urban in China

As shown in Fig. 4, the predicted values of 242 impaired health states for the rural final model were generally lower than that of the corresponding health states for the urban model, especially the predicted value of state 33321 for rural registrants is 0.124 lower than that for urban counterparts. Among all 242 impaired health states, only 13 states yield higher value in the rural model than the urban model.

The correlation coefficient between the mean observed values of all health states in the one-third validation sample and the urban model predicted values was 0.9892 which was smaller than that of the mean observed values in the validation sample and the rural model predicted values (r = 0.9937).

### Estimating a value set using rural and urban mixed sample

We merged our study sample and Liu’s urban population sample [14] (2320 respondents in total), used the same data elimination criteria and model selection criteria, and finally selected the average level of the N3 model that based on both Chinese urban and rural populations’ preferences: *U* = 1 − 0.053 − 0.100*MO2 − 0.261*MO3 − 0.104*SC2 − 0.224*SC3 − 0.080*UA2 − 0.206*UA3 − 0.101*PD2 − 0.234*PD3 − 0.080*AD2 − 0.189*AD3 − 0.019*N3 (details see Additional file 4: Appendix S4).

## Discussion

This study applied the same study design and research methodology that has been proved to be effective in the previous urban study to analyzing the preferences on health states of the rural population particularly, which is of great importance for a country with significant urban–rural differences like China. This is the first study to provide a set of weights for the 243 EQ-5D health states based on the preferences of Chinese rural registrants. The N3 model based on an OLS regression at the aggregate level was finally selected as the tariff for the rural population.

In previous valuation studies[2, 14, 26–28, 30–33], respondents were usually excluded when they had logically inconsistency in their responses, but the exclusion criteria were different, for example, strictly excluding respondents that had more than one logically inconsistent result, or only removing respondents with serious logically inconsistency (e.g., the difference in valuation was greater or equal to 0.5 [33]). Dewitt et al. reviewed commonly used exclusion criteria of logical inconsistency in the existing studies and found that it was common that respondents to have at least one logical inconsistency when the two health states be evaluated were very close to each other [34]. Therefore, it is not recommended to exclude this type of respondent [34], and our study only exclude in total of 25 respondents (2.1%) who had 4 or more logical in consistency results.

In the existing published EQ-5D-3L valuation studies using TTO data [35], 11 studies selected the main effects model (only contains 10 dummy variables) as the final model, 11 studies selected the N3 model, and the other 8 studies selected models that contain D1 term or other interaction terms. According to model performances, we selected the N3 model as the final model, which was in line with experiences from the previous urban study [14]. In existing EQ-5D-3L valuation studies which directly evaluated a relatively large amount of health states, for example, China [14], Singapore [36], and South Korea [26] studies included 97, 80, and 101 health states, respectively, estimated the valuation models based at health state aggregate level (based on mean observed value of each health state). For valuation studies evaluated health states less than 45 usually estimated the models at the individual level (based on each respondent's data) [35]. Our study involved 97 health states and also demonstrated that the model performances of aggregate level models were better than individual level models.

Our value set provides consistently lower utility values than that of the EQ-5D-3L value set developed by Zhou et al. based on the Chinese urban and rural mixed population. The EQ-5D state with the largest difference is 22332, and the difference is 0.559 (Zhou's predicted value is 0.7488 and our predicted value is 0.190. There are 33 (13.6%) and 167 (69.0%) states with a difference in the two predicted values larger than 0.4 and 0.2, respectively. Zhou’s value set indicates that the SC dimension has the greatest impact on the overall health, successively followed by MO, AD, UA, and PD [15]; while, our value set indicates that the MO dimension has the greatest impact on the overall health, and then SC, PD, UA, and AD. In addition, in Zhou’s value set, when UA and PD dimensions are at level 3, the reductions in health utility are only 0.054 and 0.041, respectively, which is barely observed in other EQ-5D-3L valuation studies. In our study, level 3 of each dimension can lead to a reduction in predicted health utilities larger than 0.1, which is in line with existing studies [35]. The main reason of the important differences between Zhou's value set and our value set is that Zhou's research used a non-standard TTO method, i.e., the observed values are lower bounded at 0 [15]. Differences in sampling, respondents’ characteristics, and modelling methods may also lead to such differences.

The comparison between rural and urban studies shows a convergence of health state valuation between the two subgroups. Nevertheless, differences do exist: (1) residents with rural registration are generally more sensitive than urban registrants when facing the same health problems, especially when faced with extreme problems associated with physical condition and self-care; (2) rural registrants are less concerned with mental problems like anxiety and depression compared to urban registrants. These differences reflect an existing urban–rural gap in preferences on health outcomes shaped by socioeconomic and institutionalized disparities, and hopefully could shed light on more efficient health policies and welfare package design with more accurate health valuations.

Compared with Liu’s EQ-5D-3L urban value set, this rural study provided generally lower utilities of 242 impaired health states suggest a tendency to trade length of life for quality of life among Chinese rural registrants. This is possibly due to two reasons. On the one hand, although covering more than 90% rural registrants since the full scale-up of deepening health system reform in 2009 [7, 8], the New Rural Cooperative Medical Scheme has a relative low financing and security level compared to the health insurance scheme for urban population [37]. While the critical illness insurance program being introduced successively, its fundraising process is actually pro-rich. In addition, other social welfare and government assistance policy for rural registrants is not as comprehensive as that for urban residents, resulting in severe dilemma for the rural of falling into or back to poverty due to illness [37–39]. Therefore, when trading off between the quality of life and the length of life, rural registrants prefer to keep healthy life to avoid potential negative impact caused by the incomprehensiveness of health and social security system. On the other hand, most rural registrants in China are manual-labor workers and self-employed whose income and life rely heavily on their health. Chinese rural people have a significant son preference, and this preference is equally strong even among rural–urban migrant women [40], which also explains to a certain extent that rural population attach importance to physical strength and economic income (requires physical health). Therefore, they highly value healthy life to ensure stable and substantial income, rather than a long length of life with illness that may result in income reduction and economic burden to their family.

Although China's urbanization process is gradually proceeding, the rural population still accounts for about half of the total population in China [7, 8], and the rural population will also exist for a long time. When making specific health decisions for rural populations [41, 42], it is more meaningful to use value sets based on the preferences of rural populations. In addition, given that some health decisions are aimed at all populations in China, this study also estimated a value set based on both Chinese urban and rural populations’ preferences.

A major limitation in this study is that we could not make accurate interpretations or comparisons. Although the same study design and research methodology as urban study was adopted in the rural study, we cannot easily identify whether the difference between rural and urban registrants is a result of genuine differences in preferences on health states due to different final models. In addition, further investigations on the Chinese urban and rural population’s preferences on health outcomes are necessary. Another limitation is that we did not conduct in-depth analysis of interviewer effects [13, 43], which requires further exploration in subsequent research.

Despite these limitations, the value set generated by the current study further supplements previous population-based studies that focused only on urban Chinese registrants by targeting the rural areas. Moreover, the research team largely comprises the researchers engaged in previous urban study whose experience have laid solid foundation for this study, and this serves as a reasonable explanation for better predictive capability of the rural model. This EQ-5D-3L value set is at present the best available EQ-5D-3L value set for health technology assessment and CUA for the rural Chinese population.

## Conclusion

Considering the difference of EQ-5D-3L health state preference between the Chinese rural and urban population, the rural tariff established in the present study should be used in health surveys of the rural population in the future.

## Supplementary Information

**Additional file 1: Appendix S1.** Parameter estimates and fit statistics of individual level models using pooled OLS and RE regression.

**Additional file 2: Appendix S2.** Parameter estimates and fit statistics of the D1 model with significant variables.

**Additional file 3: Appendix S3.** The utility of 243 health states based on Chinese rural population.

**Additional file 4: Appendix S4**. Parameter estimates and fit statistics of aggregate level models using rural and urban mixed sample.

## Data Availability

The datasets generated during and/or analysed during the current study are available from the corresponding author on reasonable request.
