# Supplementary material for: Rural population’s preferences matter: a value set for the EQ-5D-3L health states for China’s rural population
Source: Health Qual Life Outcomes. 2022 Jan 29;20:14. doi: 10.1186/s12955-022-01917-x (PMC8800217; doi:10.1186/s12955-022-01917-x)
Supplement: Supplementary file 1 — Additional file 1: Appendix S1. Parameter estimates and fit statistics of individual level models using pooled OLS and RE regression. [file 12955_2022_1917_MOESM1_ESM.docx]

**Additional file 1: Appendix S1** Parameter estimates and fit statistics of individual level models using pooled OLS and RE regression

| **Variable** | **Main Effects** | | | | |  | **N3** | | | | |  | **D1** | | | | |
| --- | --- | --- | --- | --- | --- | --- | --- | --- | --- | --- | --- | --- | --- | --- | --- | --- | --- |
|  | **OLS** | |  | **RE** | |  | **OLS** | |  | **RE** | |  | **OLS** | |  | **RE** | |
|  | **Coef.** | **SE** |  | **Coef.** | **SE** |  | **Coef.** | **SE** |  | **Coef.** | **SE** |  | **Coef.** | **SE** |  | **Coef.** | **SE** |
| Constant | 0.071 | 0.007 |  | 0.072 | 0.008 |  | 0.070 | 0.007 |  | 0.071 | 0.008 |  |  | |  |  | |
| MO2 | 0.100 | 0.005 |  | 0.103 | 0.004 |  | 0.099 | 0.005 |  | 0.103 | 0.005 |  | 0.166 | 0.007 |  | 0.172 | 0.008 |
| MO3 | 0.280 | 0.006 |  | 0.280 | 0.005 |  | 0.279 | 0.007 |  | 0.279 | 0.005 |  | 0.370 | 0.013 |  | 0.366 | 0.011 |
| SC2 | 0.101 | 0.005 |  | 0.103 | 0.005 |  | 0.101 | 0.005 |  | 0.103 | 0.005 |  | 0.169 | 0.007 |  | 0.172 | 0.008 |
| SC3 | 0.244 | 0.006 |  | 0.248 | 0.005 |  | 0.243 | 0.006 |  | 0.247 | 0.005 |  | 0.336 | 0.015 |  | 0.334 | 0.012 |
| UA2 | 0.085 | 0.005 |  | 0.084 | 0.005 |  | 0.084 | 0.006 |  | 0.084 | 0.005 |  | 0.150 | 0.007 |  | 0.151 | 0.008 |
| UA3 | 0.223 | 0.006 |  | 0.220 | 0.005 |  | 0.222 | 0.007 |  | 0.219 | 0.005 |  | 0.313 | 0.012 |  | 0.305 | 0.011 |
| PD2 | 0.110 | 0.005 |  | 0.105 | 0.005 |  | 0.109 | 0.005 |  | 0.105 | 0.005 |  | 0.175 | 0.007 |  | 0.172 | 0.009 |
| PD3 | 0.240 | 0.006 |  | 0.237 | 0.005 |  | 0.239 | 0.007 |  | 0.237 | 0.005 |  | 0.329 | 0.014 |  | 0.322 | 0.012 |
| AD2 | 0.074 | 0.006 |  | 0.074 | 0.004 |  | 0.073 | 0.006 |  | 0.074 | 0.004 |  | 0.138 | 0.007 |  | 0.140 | 0.009 |
| AD3 | 0.180 | 0.006 |  | 0.182 | 0.005 |  | 0.178 | 0.007 |  | 0.181 | 0.005 |  | 0.267 | 0.013 |  | 0.266 | 0.011 |
| N3 |  |  |  |  |  |  | 0.005^§^ | 0.007 |  | 0.003^§^ | 0.006 |  |  |  |  |  |  |
| D1 |  |  |  |  |  |  |  |  |  |  |  |  | −0.077 | 0.012 |  | −0.074 | 0.011 |
| I2 |  |  |  |  |  |  |  |  |  |  |  |  | 0.015^§^ | 0.015 |  | 0.009^§^ | 0.011 |
| I2sq |  |  |  |  |  |  |  |  |  |  |  |  | -0.001^§^ | 0.003 |  | -0.000^§^ | 0.003 |
| I3 |  |  |  |  |  |  |  |  |  |  |  |  | -0.028^†^ | 0.012 |  | −0.028 | 0.010 |
| I3sq |  |  |  |  |  |  |  |  |  |  |  |  | 0.003^§^ | 0.002 |  | 0.004 | 0.001 |
| Fit statistics |  |  |  |  |  |  |  |  |  |  |  |  |  |  |  |  |  |
| Adjusted R^2^ | 0.536 | |  | 0.536 | |  | 0.536 | |  | 0.536 | |  | 0.868 | |  |  | |
| MAE | 0.017 | |  | 0.017 | |  | 0.017 | |  | 0.017 | |  | 0.016 | |  | 0.017 | |
| RMSE | 0.282 | |  | 0.282 | |  | 0.282 | |  | 0.282 | |  | 0.282 | |  | 0.282 | |
| No. (of 97)>0.025 | 29 | |  | 28 | |  | 28 | |  | 27 | |  | 28 | |  | 28 | |
| No. (of 97)>0.05 | 3 | |  | 3 | |  | 3 | |  | 3 | |  | 2 | |  | 2 | |

P<0.01 and Heteroskedasticity-robust standard error for all regression coefficients unless otherwise stated; there are no health states that had an MAE greater than 0.1 for all models; OLS, ordinary least square; RE, random effects; Coef., coefficient; SE, standard error; MAE, mean absolute error; RMSE, root mean squared error; ^†^ 0.01≤P≤0.05, ^§^ P>0.1
