# Supplementary material for: Rural population’s preferences matter: a value set for the EQ-5D-3L health states for China’s rural population
Source: Health Qual Life Outcomes. 2022 Jan 29;20:14. doi: 10.1186/s12955-022-01917-x (PMC8800217; doi:10.1186/s12955-022-01917-x)
Supplement: Supplementary file 2 — Additional file 2: Appendix S2. Parameter estimates and fit statistics of the D1 model with significant variables. [file 12955_2022_1917_MOESM2_ESM.docx]

**Additional file 2: Appendix S2** Parameter estimates and fit statistics of the D1 model with significant variables

| **Variable** | **Aggregate Level** | | | | |  | **Individual Level** | | | | |
| --- | --- | --- | --- | --- | --- | --- | --- | --- | --- | --- | --- |
|  | **OLS** | |  | **WLS** | |  | **OLS** | |  | **RE** | |
|  | **Coef.** | **SE** |  | **Coef.** | **SE** |  | **Coef.** | **SE** |  | **Coef.** | **SE** |
| MO2 | 0.168 | 0.007 |  | 0.171 | 0.007 |  | 0.171 | 0.006 |  | 0.174 | 0.008 |
| MO3 | 0.358 | 0.010 |  | 0.351 | 0.009 |  | 0.351 | 0.008 |  | 0.351 | 0.009 |
| SC2 | 0.170 | 0.006 |  | 0.173 | 0.007 |  | 0.173 | 0.006 |  | 0.174 | 0.008 |
| SC3 | 0.321 | 0.010 |  | 0.316 | 0.009 |  | 0.316 | 0.009 |  | 0.319 | 0.009 |
| UA2 | 0.153 | 0.007 |  | 0.156 | 0.006 |  | 0.156 | 0.006 |  | 0.155 | 0.008 |
| UA3 | 0.300 | 0.008 |  | 0.295 | 0.007 |  | 0.295 | 0.007 |  | 0.291 | 0.008 |
| PD2 | 0.177 | 0.006 |  | 0.181 | 0.006 |  | 0.181 | 0.007 |  | 0.176 | 0.008 |
| PD3 | 0.315 | 0.008 |  | 0.311 | 0.008 |  | 0.311 | 0.009 |  | 0.309 | 0.009 |
| AD2 | 0.141 | 0.008 |  | 0.145 | 0.007 |  | 0.145 | 0.006 |  | 0.145 | 0.008 |
| AD3 | 0.255 | 0.009 |  | 0.251 | 0.009 |  | 0.251 | 0.008 |  | 0.254 | 0.009 |
| D1 | −0.067 | 0.007 |  | −0.071 | 0.007 |  | −0.071 | 0.007 |  | −0.072 | 0.008 |
| I3 | −0.016^†^ | 0.009 |  |  |  |  |  |  |  |  |  |
| Fit statistics |  |  |  |  |  |  |  |  |  |  |  |
| Adjusted R^2^ | 0.999 | |  | 0.999 | |  | 0.868 | |  |  | |
| MAE | 0.017 | |  | 0.017 | |  | 0.017 | |  | 0.017 | |
| RMSE | 0.024 | |  | 0.024 | |  | 0.282 | |  | 0.282 | |
| No. (of 97)>0.025 | 27 | |  | 29 | |  | 29 | |  | 28 | |
| No. (of 97)>0.05 | 2 | |  | 3 | |  | 3 | |  | 3 | |

P<0.01 and Heteroskedasticity-robust standard error for all regression coefficients unless otherwise stated; there are no health states that had an MAE greater than 0.1 for all models; OLS, ordinary least square; WLS, weighted least square; RE, random effects; Coef., coefficient; SE, standard error; MAE, mean absolute error; RMSE, root mean squared error; ^†^ P<0.1
