# Supplementary material for: Rural population’s preferences matter: a value set for the EQ-5D-3L health states for China’s rural population
Source: Health Qual Life Outcomes. 2022 Jan 29;20:14. doi: 10.1186/s12955-022-01917-x (PMC8800217; doi:10.1186/s12955-022-01917-x)
Supplement: Supplementary file 3 — Additional file 3: Appendix S3. The utility of 243 health states based on Chinese rural population. [file 12955_2022_1917_MOESM3_ESM.doc]

**Additional file 3: Appendix S3 The utility of 243 health states based on Chinese rural population**

| **State** | **Utility** |  | **State** | **Utility** |  | **State** | **Utility** |  | **State** | **Utility** |  | **State** | **Utility** |
| --- | --- | --- | --- | --- | --- | --- | --- | --- | --- | --- | --- | --- | --- |
| 11111 | 1 |  | 21222 | 0.562 |  | 11233 | 0.427 |  | 22232 | 0.321 |  | 13233 | 0.188 |
| 11112 | 0.859 |  | 12222 | 0.560 |  | 21313 | 0.427 |  | 23213 | 0.319 |  | 23313 | 0.188 |
| 11211 | 0.847 |  | 21213 | 0.558 |  | 12313 | 0.425 |  | 21323 | 0.317 |  | 33311 | 0.186 |
| 21111 | 0.832 |  | 12213 | 0.556 |  | 31311 | 0.425 |  | 33211 | 0.317 |  | 23232 | 0.185 |
| 12111 | 0.830 |  | 31211 | 0.556 |  | 21232 | 0.424 |  | 12323 | 0.315 |  | 13323 | 0.179 |
| 11121 | 0.823 |  | 11223 | 0.549 |  | 12232 | 0.422 |  | 31321 | 0.315 |  | 23322 | 0.176 |
| 11212 | 0.773 |  | 22122 | 0.545 |  | 22312 | 0.422 |  | 22322 | 0.312 |  | 23133 | 0.173 |
| 21112 | 0.758 |  | 22113 | 0.541 |  | 13213 | 0.420 |  | 13223 | 0.310 |  | 32223 | 0.171 |
| 12112 | 0.756 |  | 32111 | 0.539 |  | 11323 | 0.418 |  | 22133 | 0.309 |  | 33131 | 0.171 |
| 11122 | 0.749 |  | 21123 | 0.534 |  | 23212 | 0.417 |  | 32131 | 0.307 |  | 13332 | 0.155 |
| 21211 | 0.746 |  | 22221 | 0.533 |  | 21322 | 0.415 |  | 23222 | 0.307 |  | 31233 | 0.152 |
| 11113 | 0.745 |  | 12123 | 0.532 |  | 12322 | 0.413 |  | 11333 | 0.296 |  | 32313 | 0.150 |
| 12211 | 0.744 |  | 31121 | 0.532 |  | 21133 | 0.412 |  | 23123 | 0.295 |  | 32232 | 0.147 |
| 11221 | 0.737 |  | 11313 | 0.528 |  | 12133 | 0.410 |  | 21332 | 0.293 |  | 33213 | 0.145 |
| 22111 | 0.729 |  | 11232 | 0.525 |  | 31131 | 0.410 |  | 33121 | 0.293 |  | 31323 | 0.143 |
| 21121 | 0.722 |  | 21312 | 0.525 |  | 13222 | 0.408 |  | 12332 | 0.291 |  | 32322 | 0.138 |
| 12121 | 0.720 |  | 12312 | 0.523 |  | 22132 | 0.407 |  | 13313 | 0.289 |  | 32133 | 0.135 |
| 11311 | 0.700 |  | 13212 | 0.518 |  | 23113 | 0.405 |  | 13232 | 0.286 |  | 33222 | 0.133 |
| 11131 | 0.685 |  | 11322 | 0.516 |  | 33111 | 0.403 |  | 23312 | 0.286 |  | 23331 | 0.128 |
| 13111 | 0.678 |  | 11133 | 0.513 |  | 13123 | 0.396 |  | 32213 | 0.281 |  | 33123 | 0.121 |
| 21212 | 0.672 |  | 21132 | 0.510 |  | 22231 | 0.395 |  | 13322 | 0.277 |  | 31332 | 0.119 |
| 12212 | 0.670 |  | 12132 | 0.508 |  | 11332 | 0.394 |  | 13133 | 0.274 |  | 33312 | 0.112 |
| 11222 | 0.663 |  | 13113 | 0.506 |  | 23122 | 0.393 |  | 31223 | 0.274 |  | 33132 | 0.097 |
| 11213 | 0.659 |  | 23112 | 0.503 |  | 13312 | 0.387 |  | 23132 | 0.271 |  | 22333 | 0.092 |
| 22112 | 0.655 |  | 21231 | 0.498 |  | 22321 | 0.386 |  | 32222 | 0.269 |  | 32331 | 0.090 |
| 21122 | 0.648 |  | 12231 | 0.496 |  | 31213 | 0.384 |  | 22331 | 0.264 |  | 23233 | 0.087 |
| 12122 | 0.646 |  | 22311 | 0.496 |  | 23221 | 0.381 |  | 23231 | 0.259 |  | 33231 | 0.085 |
| 21113 | 0.644 |  | 13122 | 0.494 |  | 32212 | 0.379 |  | 32123 | 0.257 |  | 23323 | 0.078 |
| 22211 | 0.643 |  | 23211 | 0.491 |  | 13132 | 0.372 |  | 31313 | 0.253 |  | 33321 | 0.076 |
| 12113 | 0.642 |  | 21321 | 0.489 |  | 31222 | 0.372 |  | 31232 | 0.250 |  | 13333 | 0.057 |
| 31111 | 0.642 |  | 12321 | 0.487 |  | 32113 | 0.367 |  | 23321 | 0.250 |  | 23332 | 0.054 |
| 21221 | 0.636 |  | 31212 | 0.482 |  | 21331 | 0.367 |  | 32312 | 0.248 |  | 32233 | 0.049 |
| 11123 | 0.635 |  | 13221 | 0.482 |  | 12331 | 0.365 |  | 33212 | 0.243 |  | 32323 | 0.040 |
| 12221 | 0.634 |  | 22131 | 0.481 |  | 31123 | 0.360 |  | 31322 | 0.241 |  | 33223 | 0.035 |
| 11312 | 0.626 |  | 31113 | 0.470 |  | 13231 | 0.360 |  | 31133 | 0.238 |  | 31333 | 0.021 |
| 22121 | 0.619 |  | 11331 | 0.468 |  | 23311 | 0.360 |  | 32132 | 0.233 |  | 32332 | 0.016 |
| 11132 | 0.611 |  | 23121 | 0.467 |  | 32122 | 0.355 |  | 33113 | 0.231 |  | 33313 | 0.014 |
| 13112 | 0.604 |  | 32112 | 0.465 |  | 31312 | 0.351 |  | 13331 | 0.229 |  | 33232 | 0.011 |
| 11231 | 0.599 |  | 13311 | 0.461 |  | 13321 | 0.351 |  | 22233 | 0.223 |  | 33322 | 0.002 |
| 21311 | 0.599 |  | 22222 | 0.459 |  | 22223 | 0.345 |  | 32231 | 0.221 |  | 33133 | −0.001 |
| 12311 | 0.597 |  | 31122 | 0.458 |  | 23131 | 0.345 |  | 33122 | 0.219 |  | 23333 | −0.044 |
| 13211 | 0.592 |  | 22213 | 0.455 |  | 32221 | 0.343 |  | 22323 | 0.214 |  | 33331 | −0.046 |
| 11321 | 0.590 |  | 32211 | 0.453 |  | 31132 | 0.336 |  | 32321 | 0.212 |  | 32333 | −0.082 |
| 21131 | 0.584 |  | 21223 | 0.448 |  | 33112 | 0.329 |  | 23223 | 0.209 |  | 33233 | −0.087 |
| 12131 | 0.582 |  | 12223 | 0.446 |  | 21233 | 0.326 |  | 33221 | 0.207 |  | 33323 | −0.096 |
| 23111 | 0.577 |  | 13131 | 0.446 |  | 12233 | 0.324 |  | 21333 | 0.195 |  | 33332 | −0.120 |
| 22212 | 0.569 |  | 31221 | 0.446 |  | 22313 | 0.324 |  | 12333 | 0.193 |  | 33333 | −0.218 |
| 31112 | 0.568 |  | 22123 | 0.431 |  | 31231 | 0.324 |  | 31331 | 0.193 |  |  |  |
| 13121 | 0.568 |  | 32121 | 0.429 |  | 32311 | 0.322 |  | 22332 | 0.190 |  |  |  |
