# Supplementary material for: Rural population’s preferences matter: a value set for the EQ-5D-3L health states for China’s rural population
Source: Health Qual Life Outcomes. 2022 Jan 29;20:14. doi: 10.1186/s12955-022-01917-x (PMC8800217; doi:10.1186/s12955-022-01917-x)
Supplement: Supplementary file 4 — Additional file 4: Appendix S4. Parameter estimates and fit statistics of aggregate level models using rural and urban mixed sample. [file 12955_2022_1917_MOESM4_ESM.docx]

**Additional file 4: Appendix S4** Parameter estimates and fit statistics of aggregate level models using rural and urban mixed sample

| **Variable** | **Main Effects** | | | | |  | **N3** | | | | |  | **D1** | | | | |
| --- | --- | --- | --- | --- | --- | --- | --- | --- | --- | --- | --- | --- | --- | --- | --- | --- | --- |
|  | **OLS** | |  | **WLS** | |  | **OLS** | |  | **WLS** | |  | **OLS** | |  | **WLS** | |
|  | **Coef.** | **SE** |  | **Coef.** | **SE** |  | **Coef.** | **SE** |  | **Coef.** | **SE** |  | **Coef.** | **SE** |  | **Coef.** | **SE** |
| Constant | 0.057 | 0.007 |  | 0.058 | 0.007 |  | 0.053 | 0.006 |  | 0.055 | 0.006 |  |  | |  |  | |
| MO2 | 0.101 | 0.006 |  | 0.099 | 0.006 |  | 0.100 | 0.006 |  | 0.098 | 0.006 |  | 0.153 | 0.007 |  | 0.153 | 0.007 |
| MO3 | 0.265 | 0.007 |  | 0.266 | 0.007 |  | 0.261 | 0.007 |  | 0.264 | 0.007 |  | 0.337 | 0.013 |  | 0.340 | 0.012 |
| SC2 | 0.103 | 0.006 |  | 0.102 | 0.006 |  | 0.104 | 0.005 |  | 0.103 | 0.005 |  | 0.157 | 0.007 |  | 0.157 | 0.007 |
| SC3 | 0.228 | 0.006 |  | 0.229 | 0.006 |  | 0.224 | 0.007 |  | 0.228 | 0.007 |  | 0.300 | 0.013 |  | 0.304 | 0.013 |
| UA2 | 0.081 | 0.006 |  | 0.080 | 0.006 |  | 0.080 | 0.006 |  | 0.079 | 0.006 |  | 0.132 | 0.007 |  | 0.132 | 0.007 |
| UA3 | 0.212 | 0.006 |  | 0.213 | 0.006 |  | 0.206 | 0.007 |  | 0.210 | 0.007 |  | 0.282 | 0.012 |  | 0.285 | 0.013 |
| PD2 | 0.102 | 0.005 |  | 0.102 | 0.005 |  | 0.101 | 0.005 |  | 0.101 | 0.005 |  | 0.154 | 0.006 |  | 0.154 | 0.006 |
| PD3 | 0.240 | 0.006 |  | 0.242 | 0.006 |  | 0.234 | 0.007 |  | 0.240 | 0.007 |  | 0.311 | 0.012 |  | 0.314 | 0.012 |
| AD2 | 0.081 | 0.005 |  | 0.080 | 0.005 |  | 0.080 | 0.005 |  | 0.079 | 0.005 |  | 0.132 | 0.008 |  | 0.131 | 0.007 |
| AD3 | 0.195 | 0.006 |  | 0.197 | 0.006 |  | 0.189 | 0.007 |  | 0.194 | 0.007 |  | 0.264 | 0.013 |  | 0.267 | 0.012 |
| N3 |  |  |  |  |  |  | 0.019^†^ | 0.009 |  | 0.010^§^ | 0.009 |  |  |  |  |  |  |
| D1 |  |  |  |  |  |  |  |  |  |  |  |  | −0.054 | 0.011 |  | −0.056 | 0.011 |
| I2 |  |  |  |  |  |  |  |  |  |  |  |  | 0.000^§^ | 0.015 |  | 0.004^§^ | 0.016 |
| I2sq |  |  |  |  |  |  |  |  |  |  |  |  | 0.001^§^ | 0.003 |  | 0.000^§^ | 0.003 |
| I3 |  |  |  |  |  |  |  |  |  |  |  |  | −0.031^†^ | 0.013 |  | −0.034 | 0.013 |
| I3sq |  |  |  |  |  |  |  |  |  |  |  |  | 0.003^§^ | 0.003 |  | 0.004^†^ | 0.002 |
| Fit statistics |  |  |  |  |  |  |  |  |  |  |  |  |  |  |  |  |  |
| Adjusted R^2^ | 0.993 | |  | 0.995 | |  | 0.994 | |  | 0.995 | |  | 0.999 | |  | 0.999 | |
| MAE | 0.017 | |  | 0.016 | |  | 0.016 | |  | 0.016 | |  | 0.016 | |  | 0.016 | |
| RMSE | 0.023 | |  | 0.022 | |  | 0.022 | |  | 0.022 | |  | 0.022 | |  | 0.022 | |
| No. (of 97)>0.025 | 17 | |  | 16 | |  | 18 | |  | 15 | |  | 20 | |  | 20 | |
| No. (of 97)>0.05 | 5 | |  | 5 | |  | 2 | |  | 3 | |  | 2 | |  | 2 | |

P<0.01 and Heteroskedasticity-robust standard error for all regression coefficients unless otherwise stated; there are no health states that had an MAE greater than 0.1 for all models; OLS, ordinary least square; WLS, weighted least square; Coef., coefficient; SE, standard error; MAE, mean absolute error; RMSE, root mean squared error; ^†^ 0.01≤P≤0.05; ^§^ P>0.1
